# The complete chloroplast genome of the green microalgae *Dunaliella salina* strain SQ

**DOI:** 10.1080/23802359.2017.1310610

**Published:** 2017-04-13

**Authors:** Haydee Lopez, Dante Magdaleno, Jose Stephano

**Affiliations:** a Facultad de Ciencias Marinas, Universidad Autonoma de Baja California, Ensenada, Mexico;; b Facultad de Ciencias, Universidad Autonoma de Baja California, Ensenada, Mexico

**Keywords:** *Dunaliella salina*, complete chloroplast genome, microalgae

## Abstract

The complete chloroplast genome of the microalgae *Dunaliella salina* strain SQ was determined in this study. The total length of the chloroplast genome is 243,635 bp with 29.73% GC content. The genome is composed by a small single copy (SSC) region of 101,527 bp and a large single-copy region of 107,815 bp separated by two inverted repeats (IR) regions of 17,145 bp. A total of 98 genes were annotated, including 66 coding genes, 3 rRNAs, and 29 tRNAs. This complete plastid genome can be used to elucidate genetic variations in organellar genomes between *D. salina* strains.

In the past decade, green microalgae have gained interest as a source of different biocompounds such as carotenoids and other molecules of biotechnological interest. *Dunaliella salina* is a unicellular green microalgae known for been halotolerant and one of the richest sources of natural β-carotene (Lamers et al. 2008). There are well-known beneficial properties of carotenes in human health, extracts made from *D. salina* have shown anti-proliferative, anti-inflammatory, and cytotoxic activity against certain types of cancer, these properties may be due to the high content of carotenes (Chiu et al. 2016; Singh et al. 2016). Previously, the chloroplast and mitochondrial genomes of *D. salina* CCAP 19/18 has been sequenced by Smith et al. (2010) and the mitochondrial genome of *D. salina* CONC-001 has also been described (Del Vasto et al. 2015); the mitochondrial genomes present several differences between strains. Here, we report the complete chloroplast genome of *D. salina* SQ to provide information about the genetic variation between organellar genomes in *D. salina* strains.

*Dunaliella salina* was collected from San Quintin in Baja California, México (30° 32′ 13.91″ N, 116° 2′ 1.22″ W) from a hypersaline lagoon. A single cell was isolated and cultured in modified liquid medium (Feng et al. 2009) with 250 mM NaCl. Total chloroplast DNA was extracted with AxyPrep Multisource Genomic DNA Miniprep Kit (Axygen Biosciences, Union City, CA) and pure cpDNA was sequenced using Illumina MiSeq (UC, San Diego, CA). The genome was *de novo* assembled using A5 miseq pipeline (Coil et al. 2014) and Ray v2.3.2 (Boisvert et al. 2010). Annotation of the cpDNA was performed with RNAweseal (Lang et al. 2007), MFannot (Beck & Lang 2010) and tRNAscan-SE 1.21 (Schattner et al. 2005) and the results were validated with BLAST searches. The complete annotated chloroplast genome was submitted to GenBank to obtain the accession number KX530454. The complete chloroplast genome has a total length 243,635 bp with 29.73% of GC content and included two inverted repeats (IR’s) of 17,145 bp each separated by a large single-copy (LSC) region of 107,815 bp and a small single-copy (SSC) region of 101,527 bp. The genome contains 98 genes, including 66 protein-encoded genes, 29 tRNA genes and 3 rRNA genes. Five genes are situated in the IR region (*rrns*, *rrnl*, *rrn5*, *trnA*, and *trnI*). Eight genes contain introns, one with five introns (*psbA*), one with four introns (*rrnL*), two with two introns (*psbC* and *rrns*), and three contain a single intron (*atpA*, *psaB*, and *psbC*). The *psaA* gene is trans-spliced, the first two exons are situated in the LSC region while the remaining three exons are located in the SSC region and two introns were detected between them. For the phylogenetic analysis, 10 chloroplast genomes sequences from others microalgae where selected and downloaded from NCBI database and a neighbour-joining tree with 500 bootstraps was constructed using MEGA7 (Kumar et al. 2016) (Figure 1).

**Figure 1.**
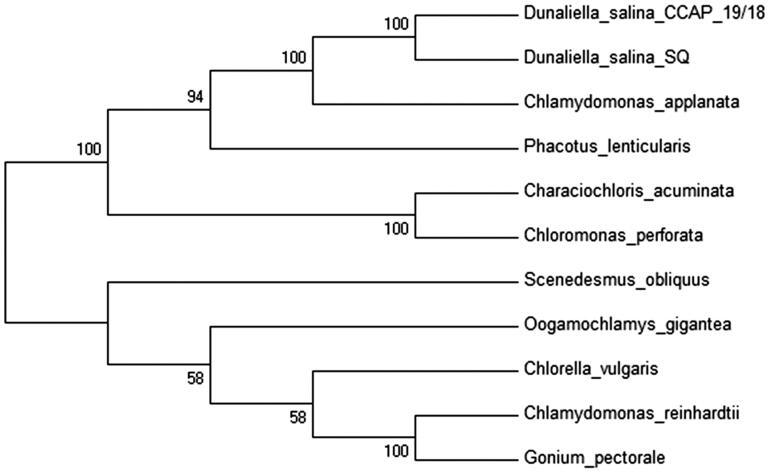
Phylogenetic tree of *D. salina* SQ and 11 microalgae chloroplast genomes including another strain of *D. salina*. The GeneBank accession numbers are listed as follow: *Characiochloris acuminata* (KT625418), *Chlamydomonas reinhard*tii (NC_005353.1), *C. applanat*a (KT625417)*, Chlorella vulgaris* (NC_001865.1), *Chloromonas perforata* (KT625416), *D. salina* (NC_016732), *D. salina* strain SQ (KX530454) (this study), *Gonium pectoral* (AP012494), *Oogamochlamys gigantean* (KT625412), *Phacotus lenticularis* (KT625422) and *Scenedesmus obliquus* (DQ396875).
